# Case Report: Oral semaglutide-associated depression in a patient with type 2 diabetes mellitus: a dose-dependent recurrence confirmed on rechallenge and managed with vortioxetine–bupropion combination therapy

**DOI:** 10.3389/fpsyt.2026.1893173

**Published:** 2026-07-24

**Authors:** Abdulrahman S. Alanazi, Basmah A. Alanazi

**Affiliations:** 1 College of Pharmacy, University of Hail, Hail, Saudi Arabia; 2 Health Holding Company, Hail Health Cluster, Hail, Saudi Arabia

**Keywords:** adverse drug reaction, bupropion, depression, dose-dependent, Naranjo scale, oral GLP-1 receptor agonist, rechallenge, semaglutide

## Abstract

**Background:**

Oral semaglutide carries a neuropsychiatric safety profile that continues to evolve. Large clinical trials have not, at the group level, demonstrated excess psychiatric adverse events—but pharmacovigilance databases and case reports have documented a signal for depression and suicidal ideation in individual patients. A dose-dependent onset with rechallenge has not, to our knowledge, been previously described for the oral formulation, though such an association cannot be established from a single observation.

**Case presentation:**

We describe a 54-year-old man with type 2 diabetes and no psychiatric history who developed moderately severe major depression (Patient Health Questionnaire-9 [PHQ-9]: 18; Hamilton Depression Rating Scale 17 items [HDRS-17]: 22) within 2 weeks of escalating oral semaglutide from 3 to 7 mg daily. Vortioxetine 10 mg combined with bupropion XL 150 mg produced remission within 4 weeks (PHQ-9: 6). On structured rechallenge, depression returned when the dose was re-escalated to 7 mg (PHQ-9: 17; HDRS-17: 20). Permanent discontinuation was followed by sustained remission.

**Conclusion:**

As far as we are aware, this is the first reported case of a dose-related, rechallenge-associated depressive episode tied in time to oral semaglutide and treated with a vortioxetine–bupropion combination. A Naranjo score of 7 puts the relationship in the probable range, though that reading is held back by the usual limits of a single case and by an incomplete washout before rechallenge. The step from 3 to 7 mg may be a point of raised psychiatric risk in vulnerable patients, and watching mood during titration looks like a sensible precaution.

## 1 Introduction

Semaglutide is a once-daily oral or once-weekly injectable glucagon-like peptide-1 receptor agonist (GLP-1 RA) with regulatory approval for type 2 diabetes mellitus (T2DM) and, at higher doses, for obesity management. The oral formulation—Rybelsus, approved by the FDA in 2019—follows a standard titration: 3 mg daily for the first 4 weeks, then 7 mg, with a maximum of 14 mg for those requiring further glycaemic control (1), GLP-1 receptors are not confined to the pancreas or gut. They are expressed in the prefrontal cortex, amygdala, hippocampus, and hypothalamus—brain regions that sit at the centre of mood regulation. Their activation alters the release of serotonin, dopamine, and glutamate, all of which play established roles in the pathophysiology of major depressive disorder (MDD) (2).

The psychiatric safety of semaglutide has been under regulatory scrutiny for some time. A *post hoc* analysis of the STEP 1–5 trials by Wadden et al. found that depressive symptoms serious enough to require clinical evaluation occurred in 2.8% of semaglutide-treated patients versus 4.1% with placebo—a finding that, at first glance, suggests a protective rather than harmful effect at the population level (3).

The pharmacovigilance data complicate that picture. A retrospective analysis of the FDA’s Adverse Event Reporting System (FAERS) identified disproportionality signals for semaglutide in both depression (reporting odds ratio [ROR]: 1.87; 95% confidence interval [CI]: 1.60–2.20) and suicide or self-injury events (ROR: 1.73; 95% CI: 1.46–2.04), concentrated in the 18–64 age range (4). Population-level reassurance and individual-level risk are not the same thing, and this tension runs through the literature.

The first published case series linking semaglutide to depression was reported by Li et al.—two patients who developed depressive symptoms around a month after starting subcutaneous semaglutide, both resolving on discontinuation. Neither involved the oral formulation, a dose-dependent pattern, nor a rechallenge (5).

We report a case that differs from those on all three counts: depression emerging specifically at the 7 mg dose (not the 3 mg), recurring at the identical titration step on rechallenge, and responding to a rationally selected vortioxetine–bupropion combination whose design addressed the hypothesised dopaminergic mechanism. The patient had no prior psychiatric history. We present this as a hypothesis-generating observation; although the available causality instruments support a probable association, a single case cannot establish causation, and the alternative explanations and methodological limitations are discussed in detail below. This case report has been prepared and is reported in accordance with the CAse REport (CARE) guidelines (17).

## 2 Case presentation

### 2.1 Patient background and baseline assessment

A 54-year-old Saudi Arabian man attended the outpatient diabetes clinic for glycaemic review. He had lived with T2DM for 8 years, managed on metformin 1 g twice daily, alongside hypertension treated with amlodipine 5 mg once daily. His BMI was 31.4 kg/m^2^, glycated haemoglobin (HbA1c) 8.6%, and fasting plasma glucose (FPG) 11.2 mmol/L. There was no personal or family psychiatric history, no previous psychotropic medications, no alcohol or substance use, and no recent life stressors he could identify. A baseline Patient Health Questionnaire-9 (PHQ-9) of 3 and GAD-7 of 2 confirmed minimal symptoms in both mood and anxiety domains.

### 2.2 Initiation of oral semaglutide and dose escalation

We added oral semaglutide 3 mg daily to his metformin. He took it well—a little nausea in the first week, nothing more—and dropped 1.8 kg over the month. The week-4 review was already on the calendar as the dose-escalation visit, and since that is the labelled point for moving from 3 to 7 mg, we used it to recheck his mood before stepping up rather than because anything had prompted concern; his PHQ-9 was 5. At the time, both he and the clinician read that small uptick from baseline as the fatigue of dose adjustment and the gut settling in, so titration went ahead as labelled.

At week 4, semaglutide was escalated to 7 mg. By approximately week 6—2 weeks after the dose change—something had clearly shifted. The patient described an emotional state entirely unlike anything he had experienced before: pervasive low mood, loss of interest in activities he had always found meaningful, profound fatigue, hypersomnia pushing sleep beyond ten hours most nights, psychomotor slowing, difficulty concentrating, and passive thoughts of death without any plan or intent. There was no identifiable precipitant. He could not point to any life event, relationship change, or occupational stressor that might explain it.

### 2.3 Psychiatric assessment and diagnosis

Formal psychiatric assessment confirmed the clinical picture. PHQ-9 had reached 18—moderately severe depression—and Hamilton Depression Rating Scale 17 items (HDRS-17) was 22, meeting DSM-5 criteria for a moderate-to-severe major depressive episode. Investigations, including thyroid function (TSH: 2.1 mIU/L), full blood count, renal function, electrolytes, and vitamin B12, came back unremarkable. The temporal link to dose escalation was notable: the patient had been stable on 3 mg, he had escalated to 7 mg, and within 2 weeks, he met criteria for a moderate-to-severe episode.

Before pinning the episode on semaglutide, we worked through the alternatives. A primary depressive disorder unrelated to the drug was possible, but it fit poorly: the patient had no personal or family psychiatric history, there was no life event or stressor to point to, and the symptoms sat right on top of the dose escalation. Organic and metabolic causes were checked and ruled out—thyroid function was normal, the full blood count gave no sign of anaemia or infection, renal function and electrolytes were unremarkable, and B12 was not deficient. The harder question was whether what looked like depression was really a somatic by-product of GLP-1 treatment: appetite suppression, steady weight loss, gastrointestinal upset, and fatigue can all read as low mood, or worsen it. A few things argued otherwise. The gastrointestinal symptoms had been mild and confined to the first week. The dominant features were psychological rather than physical—deep anhedonia, loss of interest in things that had always mattered to him, passive thoughts of death—and those are not well explained by a smaller appetite or a few lost kilograms. And the patient drew the distinction himself, describing the state as something other than being tired. That said, the somatic and metabolic effects of these drugs can blur into, feed, or magnify a depressive picture, and one case cannot fully separate the threads. Substance-induced mood disorder and bipolar depression were considered, too; neither was supported by the history.

We did weigh the obvious alternative of dropping semaglutide back to 3 mg, or slowing the climb, before reaching for an antidepressant. During the acute episode, we chose not to. The depression was moderately severe and came with passive suicidal ideation (PHQ-9: 18, HDRS-17: 22), which on its own called for prompt antidepressant treatment, whatever the trigger turned out to be—this was not a situation for watchful waiting tied to dose tinkering. At that moment, the dose–symptom link was still only a suspicion; it became something we could actually test only later, which is precisely why the structured downtitration and rechallenge were done. Semaglutide stayed at 7 mg through the early part of management, giving us a steady background against which to read the antidepressant response, and the deliberate discontinuation and rechallenge that followed let us probe the dose–response relationship head-on.

### 2.4 Antidepressant management

Vortioxetine 10 mg once daily was selected as the primary antidepressant. The rationale went beyond convenience: vortioxetine acts through multiple serotonergic mechanisms simultaneously—serotonin transporter (SERT) inhibition alongside 5-HT1A agonism, 5-HT3 and 5-HT7 antagonism, and 5-HT1B partial agonism—giving it a pharmacological breadth suited to a drug-induced depression where the precipitating mechanism was unlikely to be purely serotonergic (6).

Bupropion XL 150 mg once daily was added specifically to address the dopaminergic dimension. Emerging preclinical and pharmacogenomic evidence suggests that semaglutide suppresses dopamine release in the nucleus accumbens and blunts ventral tegmental area responses to reward—a mechanism that maps directly onto the anhedonia and amotivation dominating this patient’s presentation. Bupropion’s norepinephrine–dopamine reuptake inhibition (NDRI) mechanism was chosen to counteract precisely that suppression (7, 8).

Standard practice for a first moderate depressive episode is to see whether a single antidepressant works before combining two, so starting vortioxetine and bupropion together needs an explanation. The picture was dominated by anhedonia, flat motivation, and psychomotor slowing—a hedonic, dopaminergic cluster sitting on top of what we suspected was a drug-induced mechanism. A purely serotonergic agent seemed unlikely, on mechanistic grounds, to cover that whole picture. The passive suicidal ideation also made us want a faster, more complete response than monotherapy typically delivers. And the dopaminergic case for bupropion was tailored to this particular episode rather than a generic add-on. We are not claiming this was the only correct route. A monotherapy-first approach would have been just as defensible, arguably more conventional, and because we used both drugs at once, we cannot say how much each one contributed to the recovery.

The pharmacokinetic interaction between these two agents required explicit management. Bupropion is a potent CYP2D6 inhibitor; coadministration approximately doubles vortioxetine plasma exposure. The vortioxetine starting dose of 10 mg was deliberately chosen with that interaction in mind—an intentionally lower dose to account for the expected elevation in exposure—and the patient was monitored closely for serotonergic excess.

By week 12—4 weeks into the antidepressant regimen—PHQ-9 had fallen to 6 and HDRS-17 to 9. Anhedonia had resolved, passive suicidal ideation had cleared, and psychomotor function had normalised. Meanwhile, weight continued to decline (− 3.4 kg total), and HbA1c had improved to 7.2%, confirming that semaglutide was maintaining its glycaemic effect throughout.

### 2.5 Discontinuation and structured rechallenge

After a shared decision-making discussion, semaglutide was stopped at week 16 to test mood stability independent of the drug. Over the following 2 weeks, PHQ-9 held steady at 5—but FPG climbed back to 9.8 mmol/L, underscoring the real glycaemic contribution of semaglutide and creating a genuine clinical dilemma: the drug appeared to be both causing harm and providing benefit.

To settle that dilemma, we set up a structured rechallenge with safeguards built in. The patient was part of the decision and gave written informed consent after we talked through the risks, including the chance that the depression would come back. Several things lowered the danger. He was in remission when we began (PHQ-9: 5), his vortioxetine and bupropion stayed in place throughout, monitoring was tightened to weekly PHQ-9 and HDRS-17 scores, and we agreed beforehand that any clear slide, above all a return of suicidal ideation, would stop the drug at once. Semaglutide went back in at 3 mg with weekly checks. For 4 weeks, it looked just as it had the first time: PHQ-9 holding at 5, HDRS-17 at 7, nothing of concern. At week 22, at the same titration point as before, the dose was then stepped up to 7 mg. PHQ-9 was already climbing before the escalation finished (12 at week 21), and by week 24, it had reached 17 with HDRS-17 back at 20. Following the plan we had set, semaglutide was stopped there rather than held or pushed higher.

Semaglutide was permanently discontinued at week 24. By week 28, PHQ-9 had settled to 4 and HDRS-17 to 6, with the patient remaining on vortioxetine and bupropion. Glycaemic management was switched to empagliflozin 10 mg daily; FPG stabilised at 8.1 mmol/L at the most recent follow-up.

## 3 Clinical timeline

**Figure 1:**
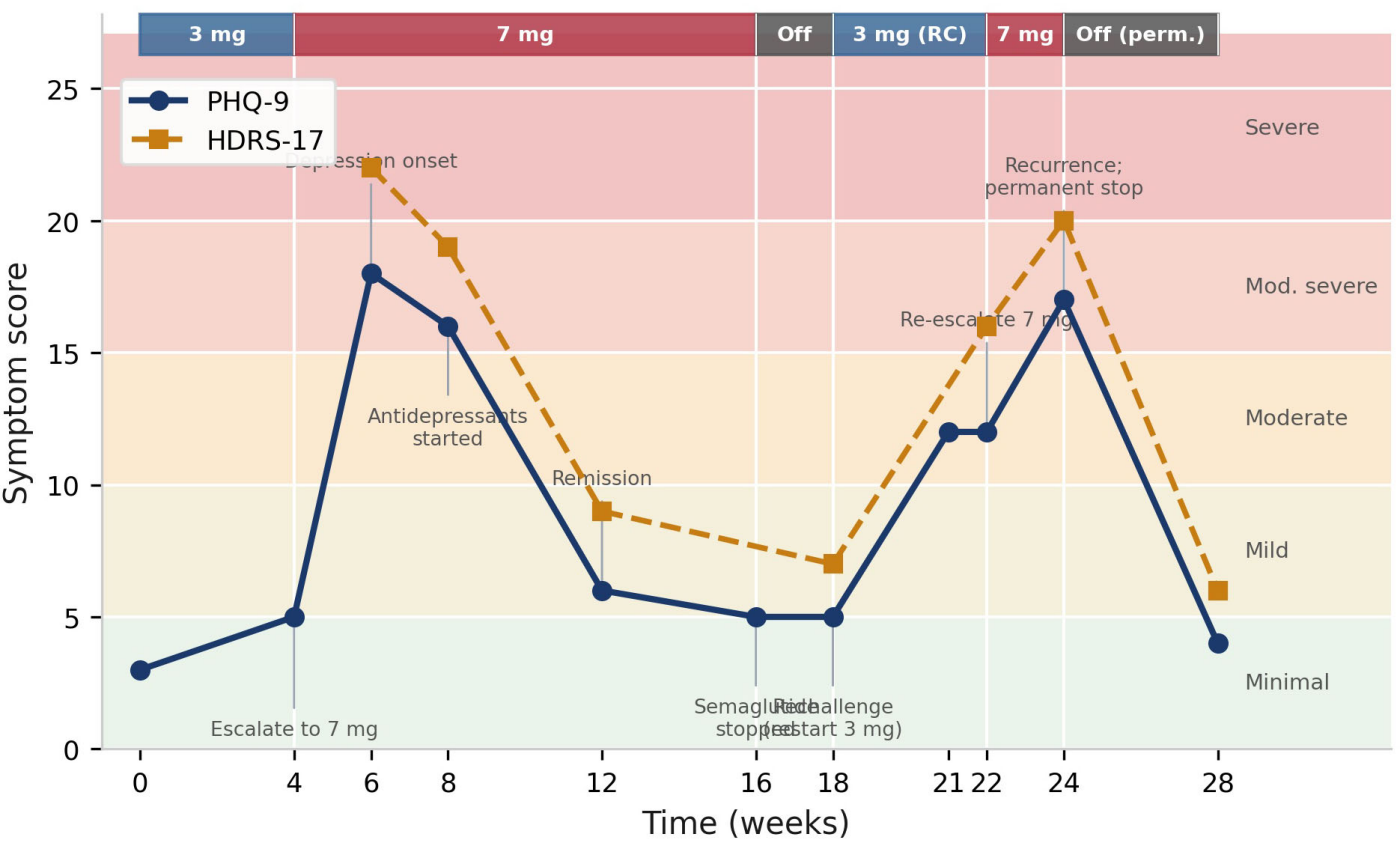
Longitudinal course of depressive symptoms (PHQ-9 and HDRS-17) in relation to oral semaglutide dose. The coloured strip indicates the semaglutide dose phase at each time point; horizontal bands denote PHQ-9 severity categories. Symptom scores rose into the moderately severe range within 2 weeks of escalation to 7 mg, remitted following initiation of vortioxetine–bupropion combination therapy, and recurred at the equivalent 7-mg titration step during the structured rechallenge before resolving after permanent discontinuation. PHQ-9, Patient Health Questionnaire-9; HDRS-17, Hamilton Depression Rating Scale, 17-item; RC, rechallenge. The 2-week washout preceding rechallenge was shorter than required for complete clearance, given semaglutide’s approximately 1-week half-life; therefore, the rechallenge phase should be interpreted in light of the limitations discussed in Section 5.8.

The full clinical course is summarised graphically in **Figure 1** and in detail in **Table 1**, which together track dose titrations, interventions, and corresponding psychiatric assessments across the episode of care.

**Table 1:** Clinical timeline of oral semaglutide-associated depression, antidepressant management, and rechallenge.

Timepoint	Event	Clinical details	Assessment/action
Baseline (week 0)	Oral semaglutide initiated	3 mg once daily; T2DM on metformin 1 g BD; BMI: 31.4 kg/m^2^; HbA1c: 8.6%; no psychiatric history	PHQ-9: 3; GAD-7: 2; FPG: 11.2 mmol/L
Week 4	Dose uptitration to 7 mg	Increased per label; GI tolerability acceptable; weight: − 1.8 kg	PHQ-9: 5 (minimal); fatigue attributed to dose change
Week 6	Depressive syndrome onset	Progressive low mood, anhedonia, profound fatigue, hypersomnia, psychomotor slowing, passive SI without intent. Semaglutide continued at 7 mg	PHQ-9: 18 (moderately severe); HDRS-17: 22
Week 8	Antidepressants initiated; semaglutide continued	Vortioxetine 10 mg + bupropion XL 150 mg commenced. Semaglutide 7 mg continued. CYP2D6 interaction counselled	PHQ-9: 16; HDRS-17: 19
Week 12	Antidepressant response confirmed	Full resolution of anhedonia and SI. Semaglutide 7 mg throughout	PHQ-9: 6 (mild); HDRS-17: 9; weight: − 3.4 kg; HbA1c: 7.2%
Week 16	Semaglutide discontinued; rechallenge planned	Shared decision to discontinue and assess mood stability. Stable on semaglutide for 2 weeks	PHQ-9: 5; FPG rising to 9.8 mmol/L
Week 18 (rechallenge)	Oral semaglutide restarted at 3 mg	Close weekly psychiatric monitoring. Written informed consent obtained	PHQ-9: 5; HDRS-17: 7
Week 22 (rechallenge + 4)	Uptitrated to 7 mg	PHQ-9 rising before uptitration (score 12 at week 21); escalated to 7 mg per protocol	PHQ-9: 12 (moderate); HDRS-17: 16—worsening
Week 24	Depression recurrence confirmed; semaglutide permanently stopped	Same temporal pattern and severity as the initial episode. Semaglutide permanently discontinued	PHQ-9: 17; HDRS-17: 20; WHO-UMC: probable
Week 28 (follow-up)	Sustained remission on antidepressants	Vortioxetine + bupropion continued. Switched to empagliflozin 10 mg for glycaemic management	PHQ-9: 4; HDRS-17: 6; FPG: 8.1 mmol/L

*PHQ-9*, Patient Health Questionnaire-9; *HDRS-17*, Hamilton Depression Rating Scale 17 items; *SI*, suicidal ideation; *FPG*, fasting plasma glucose. Scores: 0–4: minimal, 5–9: mild, 10–14: moderate, 15–19: moderately severe, 20–27: severe.

## 4 Causality assessment—naranjo adverse drug reaction probability scale

A Naranjo score of 7 places this reaction in the probable category (**Table 2**). The World Health Organization Uppsala Monitoring Centre (WHO-UMC) causality classification independently reaches the same conclusion—Probable—on the basis of a plausible temporal relationship between dose escalation and symptom onset, improvement on drug withdrawal, recurrence on rechallenge, the absence of an alternative plausible explanation on systematic differential assessment, and an apparent dose–response relationship at the 3- to 7-mg transition. We retain the score of 7 because each contributing item is individually defensible; however, two qualifications should temper its interpretation. First, the score derives from a single patient and is not generalisable. Second, and more importantly for the rechallenge items, oral semaglutide has a terminal half-life of approximately 1 week, so the 2-week washout preceding rechallenge would have left an estimated quarter of the previous exposure in the patient’s system. The deterioration observed on re-escalation to 7 mg is therefore confounded by residual drug accumulation and cannot be cleanly attributed to the re-escalation step alone; the same caveat applies to the apparent pre-escalation rise in PHQ-9 at week 21. These limitations do not negate the temporal and dose-related signal, but they do mean the rechallenge should be read as supportive rather than confirmatory evidence, and the Naranjo score interpreted with corresponding caution.

**Table 2:** Naranjo adverse drug reaction probability scale—oral semaglutide-associated depression.

Naranjo Criterion	Yes	No	Score
1. Are there previous conclusive reports on this reaction?	+ 1	0	0
2. Did the adverse event appear after the drug was administered?	+ 2	− 1	**+ 2**
3. Did the adverse event improve when the drug was discontinued?	+ 1	0	**+ 1**
4. Did the adverse event reappear when the drug was readministered?	+ 2	− 1	**+ 2**
5. Are there alternative causes that could have caused the reaction?	− 1	+ 2	0
6. Did the reaction reappear when a placebo was given?	− 1	+ 1	0
7. Was the drug detected in blood at a toxic concentration?	+ 1	0	0
8. Was the reaction more severe when the dose was increased?	+ 1	0	**+ 1**
9. Did the patient have a similar reaction to the same drug previously?	+ 1	0	0
10. Was the adverse event confirmed by objective evidence?	+ 1	0	**+ 1**
Total Naranjo score			**7**

Score 7 = Probable adverse drug reaction (definite ≥ 9; probable 5–8; possible 1–4; doubtful ≤ 0). Shaded cells = scored items.

Bold values indicate criteria contributing to the total Naranjo score.

## 5 Discussion

The result of this case turns on a Naranjo score of 7—a probable causal link between oral semaglutide and the depressive episode, with the WHO-UMC system classifying it similarly. Four things push the score up: the symptoms tracked dose escalation in time, they eased when the drug came off, they came back on rechallenge, and there was an apparent dose–response across the 3- to 7-mg step. One thing weighs against it, which is the incomplete washout before rechallenge (Section 5.8). A probable-category attribution is what makes the case clinically useful, and the rest of this discussion follows from it. We look at what the dose-dependent timing says about when risk peaks during titration (Section 5.1), the mechanism that makes the link biologically plausible (Section 5.2), what the treatment response tells us about management (Section 5.3), where the case fits against existing pharmacovigilance data (Section 5.4), and what it means at the bedside (Section 5.5). None of this proves causation. A single report, however carefully scored, cannot—and the Naranjo result is best read as a disciplined estimate of probability, not a verdict (5, 9).

### 5.1 Dose-dependent temporal pattern

The depression did not show up at 3 mg. It came after the move to 7 mg—the kind of detail that is easy to walk past in a busy clinic but may matter here. When we rechallenged, the 3-mg phase again went by without trouble, and the symptoms returned only as he approached 7 mg. We have not found a dose-related pattern like this reported before for oral semaglutide, and Li et al. did not describe one in their subcutaneous cases. Still, we read it carefully: as Section 5.8 spells out, the short 2-week washout leaves the drug on board and confounds the rechallenge, so this points toward a dose–response relationship rather than proving one. Taken with that caveat, it fits the idea that central GLP-1 receptor activity crosses an exposure threshold beyond which dopaminergic reward circuitry is damped enough to produce real depressive symptoms in vulnerable people (2, 10).

### 5.2 Neurobiological mechanism

GLP-1 receptors in the hypothalamus, prefrontal cortex, and amygdala modulate serotonin, dopamine, and glutamate release. The dopaminergic suppression of the nucleus accumbens by semaglutide—well-documented in preclinical models—appears to underlie the motivational and hedonic deficits that dominate the clinical picture of drug-induced depression in this context. A 2025 genetic pharmacology study went further, identifying individuals with low baseline dopamine function as specifically vulnerable to GLP-1 agonist-induced depression and suicidal ideation (2, 10, 11).

That reading of the mechanism is what drove the treatment choice. If the trouble is dopaminergic suppression, then the natural partner for a serotonergic drug is one that puts dopaminergic tone back, which is what bupropion does, through NDRI activity in the mesolimbic pathway. We cannot say for certain whether the recovery owed more to bupropion’s dopaminergic push, to vortioxetine’s multimodal serotonergic action, or to the two working together. The response held up—remission inside 4 weeks, and steady through the rechallenge, which is at least a sign we were aiming at the right target (12).

### 5.3 Rationale and pharmacokinetics of vortioxetine–bupropion combination

Picking vortioxetine over a standard SSRI or SNRI was a considered choice. A drug-induced depression may not run on a mainly serotonergic mechanism, so an agent with a wider receptor footprint (SERT inhibition plus 5-HT1A agonism, 5-HT3 and 5-HT7 antagonism, and 5-HT1B partial agonism) looked better matched to the uncertainty about what was actually driving the episode. Combining it with bupropion does raise a CYP2D6 problem: bupropion roughly doubles vortioxetine exposure, so the dose has to be managed. Prescribing guidance caps vortioxetine at 10 mg alongside a potent CYP2D6 inhibitor, and that is what we did (7, 8). Remission came within 4 weeks on both the first episode and the rechallenge, which suggests the combination, at these doses and with close monitoring, was both safe and effective against the mechanism that seems to sit behind this presentation.

### 5.4 Comparison with published literature

Group-level data do not rule out harm to an individual, and this case is a plain example of the gap. The *post hoc* analysis of the STEP programme reported depressive symptoms in 2.8% of semaglutide patients against 4.1% on placebo—a figure that looks reassuring until you notice the population was mostly nondiabetic and on subcutaneous 2.4 mg, which tells us nothing about lower doses of the oral drug in people with T2DM (3). The oral route behaves differently in pharmacokinetic terms, and patients with T2DM may bring their own baseline neurobiological vulnerabilities.

The FAERS signal (a reporting odds ratio of 1.87 for depression) and the adjusted ratio of 1.70 for depressed mood disorders from VigiBase both fit a risk that shows up at the level of the individual patient, even when the randomised trials, looking at groups, do not see it (4, 13). These disproportionality signals raise hypotheses rather than settle them, and they carry the usual reporting and confounding caveats; what our case adds is one dose-linked rechallenge observation alongside that pharmacovigilance picture—a complement to it, not a confirmation.

A propensity-matched cohort study in 162,253 patients found a 195% higher risk of major depression with GLP-1 RA treatment compared with matched controls (14). The confounders in observational data are difficult to fully exclude, but the magnitude of that signal—combined with the pharmacovigilance database findings and our rechallenge data—makes it hard to dismiss.

### 5.5 Clinical implications

A few practical lessons come out of this case. It is worth getting a validated mood baseline before starting oral semaglutide; a PHQ-9 takes about three minutes and can genuinely shift a decision. When the dose goes up, especially across the 3- to 7-mg step, check the mood actively rather than waiting for the patient to raise it. A depression that tracks the dose ought to put semaglutide on the list of possible causes even when there is no psychiatric history at all. If semaglutide-associated depression does happen, the vortioxetine–bupropion pairing used here, with vortioxetine deliberately held at 10 mg to allow for the CYP2D6 interaction, is a pharmacologically sensible option that brought remission twice in this patient. And when semaglutide is doing real cardiometabolic good, a careful, closely monitored rechallenge can resolve the dilemma instead of leaving it hanging.

### 5.6 Interdisciplinary clinical pharmacist collaboration

This case was managed jointly by the treating physician and a clinical pharmacist, and that arrangement mattered. It shaped how the reaction was caught and how it was eventually unwound. The patient occupied the kind of clinical territory where drug-related problems tend to cluster: an active somatic illness needing glucose-lowering treatment, a new depressive disorder needing an antidepressant, and the two regimens running at once. The pharmacist’s contribution was practical. The temporal link between each semaglutide step-up and the patient’s mood dip was read as a possible drug effect rather than waved through as a coincidental low mood. The Naranjo assessment was worked through formally. The CYP2D6 interaction between bupropion and vortioxetine was anticipated and quantified ahead of prescribing, which is why vortioxetine was started low. The monitoring intervals and stopping rules that later governed the discontinuation and rechallenge were set in advance. The physician carried the diagnosis and the overall care; the pharmacist concentrated on interactions, causality, and drug-safety surveillance. A dose-dependent signal this subtle is easy to miss, and splitting the work this way is largely why it was not.

What happened here lines up with a wider literature showing that bringing clinical pharmacists into psychiatric care helps surface and resolve drug-related problems, especially when somatic illness sits alongside the mental disorder. Stuhec and Batinic, writing up case experience from a mental health hospital, found that pharmacists working within transition-of-care and medication reconciliation picked up omitted drugs and medication errors that would otherwise have slipped through, among them diabetes treatment left off at admission (15). Their later pre–post-study of daily interdisciplinary ward rounds in a psychiatric hospital put numbers to it: around 1.5 pharmacist recommendations per patient, an acceptance rate near 89% at discharge, and better adherence to guidelines for the somatic comorbidities (16). Our single patient is a one-case version of the same point those service-level studies make: when a metabolic drug and a psychotropic regimen have to be run together, a structured physician–pharmacist partnership makes the medication safer. So when GLP-1 receptor agonists are prescribed to patients who have, or are prone to, mental disorders, it seems worth involving a clinical pharmacist in interaction management and pharmacovigilance—and the model deserves a proper prospective trial aimed specifically at GLP-1-related neuropsychiatric monitoring.

### 5.7 Ethical considerations of the rechallenge

Deliberately putting a patient back on a drug we suspected had driven a moderately severe depression with passive suicidal thoughts raises real ethical questions, and they deserve a straightforward answer. We do not pretend that the rechallenge was obviously the right thing to do. What pushed us toward it was a genuine bind: off semaglutide, the patient’s fasting glucose had climbed to 9.8 mmol/L, and the drug had clearly helped both his glucose and his weight, so whether it could be restarted safely was a live clinical question, not an academic one. We did it with the patient in remission, with his antidepressants kept running, with weekly psychiatric checks, and with an agreement set out beforehand to stop the moment things slipped—and we stopped it, as soon as symptoms returned and before any suicidal ideation came back. Consent was informed and in writing. Two honest caveats remain. The rechallenge rested on a clinical decision and the patient’s consent; it never went to a research-ethics committee, which would have been the better route for any planned re-exposure of this sort. Looking back, with empagliflozin having since proved a safe and effective substitute, the rechallenge was probably avoidable—a similar dilemma next time could reasonably be settled by switching drugs rather than re-exposing the patient. We lay the reasoning out plainly so readers can judge the risk and benefit for themselves.

### 5.8 Limitations

This report has clear limits. It is a single uncontrolled case, so it can raise a hypothesis but not settle one—no firm causal claim about oral semaglutide and depression can rest on one patient. The biggest methodological weakness is the rechallenge. Oral semaglutide has a terminal half-life of roughly a week, and the 2-week gap before we restarted it was not long enough to clear the drug; approximately a quarter of the earlier exposure was probably still on board when retitration began. That means the worsening on re-escalation is tangled up with drug accumulation and cannot be put down to the dose increase alone, which softens the rechallenge-dependent Naranjo items and the dose-dependence story along with them. There is a diagnostic caveat too: we screened for organic and psychosocial causes and worked through a differential, but the overlap between depression and the somatic and metabolic effects of GLP-1 therapy—less eating, weight loss, gastrointestinal upset, fatigue—cannot be wholly excluded as a contributor. The treatment choices add their own ambiguity, since starting both antidepressants at once and keeping semaglutide running early on means the recovery cannot be split cleanly between the two drugs, natural fluctuation, or the change in trigger. We also never tested dose reduction or a slower titration as a first move during the acute episode. The lack of formal ethics-committee review of the rechallenge, already noted, is a governance limitation in its own right. None of this erases the temporal, dose-related signal—but it does mean the signal should be read with caution and treated as hypothesis-generating.

## 6 Conclusion

This report describes a depressive episode that tracked oral semaglutide in time and dose, in a man with type 2 diabetes and no psychiatric history, who came back on rechallenge and lifted with a vortioxetine–bupropion combination. A Naranjo score of 7 and a WHO-UMC Probable rating point to a probable link, but this is one case with an incomplete washout before rechallenge, so it raises a hypothesis rather than proving cause. What it does suggest is that the 3- to 7-mg step may be a window of raised psychiatric risk in susceptible people—plausibly through GLP-1 effects on mesolimbic dopamine—and that a quick, validated mood check folded into the titration schedule would be a sensible precaution. Resolution of this question will require larger pharmacovigilance studies and prospective investigations. In the meantime, it appears reasonable for clinicians to consider semaglutide as a possible contributor whenever depressive symptoms emerge during dose escalation.

## 7 Patient perspective

The patient provided written consent for publication and expressed a specific wish that his experience be shared with clinicians. He described the depressive episode as categorically different from any low mood he had previously experienced—sudden, severe, and entirely unrelated to his life circumstances. He stated that, without antidepressant treatment and discontinuation of the higher dose, he did not believe he would have recovered independently.

## Data Availability

The original contributions presented in the study are included in the article/supplementary material. Further inquiries can be directed to the corresponding author.
